# Anaphylaxis Associated With Allergen Specific Immunotherapy, Omalizumab, and Dupilumab: A Real World Study Based on the US Food and Drug Administration Adverse Event Reporting System

**DOI:** 10.3389/fphar.2021.767999

**Published:** 2021-10-22

**Authors:** Sainan Bian, Pingping Zhang, Lisha Li, Zixi Wang, Le Cui, Yingyang Xu, Kai Guan, Bin Zhao, Zhuanggui Chen

**Affiliations:** ^1^ Department of Allergy, Peking Union Medical College Hospital, Peking Union Medical College, Chinese Academy of Medical Sciences, Beijing, China; ^2^ Beijing Key Laboratory of Precision Medicine for Diagnosis and Treatment of Allergic Disease, Peking Union Medical College, Beijing, China; ^3^ National Clinical Research Center for Dermatologic and Immunologic Diseases (NCRC-DID), Beijing, China; ^4^ Department of Pediatrics, The Third Affiliated Hospital of Sun Yat‐Sen University, Guangzhou, China; ^5^ Department of Allergy, The Third Affiliated Hospital of Sun Yat‐Sen University, Guangzhou, China; ^6^ Department of Pharmacy, Peking Union Medical College Hospital, Peking Union Medical College, Chinese Academy of Medical Sciences, Beijing, China

**Keywords:** anaphylaxis, allergen specific immunotherapy, omalizumab (xolair), dupilumab, FDA adverse event reporting system (FAERS)

## Abstract

**Background:** Real-world studies on the allergen specific immunotherapy (AIT), omalizumab, and dupilumab associated anaphylactic events are limited. We aimed to analyze the characteristics of drug associated anaphylaxis, and to compare the differences among different drugs.

**Methods:** A disproportionality analysis and Bayesian analysis were used in data mining to identify suspected anaphylaxis associated with AIT, omalizumab, and dupilumab based on the Food and Drug Administration (FDA) Adverse Event Reporting System (FAERS) from January 2004 to March 2021. Demographic information, time interval to onset, and death rates of AIT, omalizumab, and dupilumab associated anaphylaxis were also analyzed.

**Results:** Totally 9,969 anaphylactic events were identified. Reports of AIT, omalizumab, and dupilumab associated anaphylactic events were 64, 7,784, and 2,121, respectively. AIT had a high reporting odds ratio (ROR) of 5.03 [95%confidental interval (CI) 3.69–6.85], followed by omalizumab (ROR 2.24, 95% CI 2.18–2.29), and dupilumab had a negative signal for anaphylaxis. In children, most anaphylactic reactions (68%) were reported in the 12–17-year-old group. More reports of anaphylaxis related to AIT were in boys (73%), while more reports of anaphylaxis related to omalizumab (63%) and dupilumab (58%) were in girls. Most symptoms occurred on the day of drug initiation. The death rate of AIT related anaphylaxis was the lowest (0%), the death rate of omalizumab was 0.87%, while the death rate of dupilumab was 4.76%. No significant differences were observed among these drugs.

**Conclusion:** AIT and omalizumab had a positive signal for anaphylaxis, while dupilumab had a negative signal for anaphylaxis. Patients should be strictly monitored after administration of AIT and also biologics. It also gives us a suggestion for choosing a combined biologics with AIT when the risk of anaphylaxis was considered.

## Introduction

Allergic diseases including allergic rhinitis (AR) and asthma are becoming a worldwide chronic health problem in recent years (Brożek et al., 2017). Treatment of AR and asthma includes avoidance of allergens, drugs to control symptoms, allergen immunotherapy (AIT), and recently biologics (Roberts et al., 2018; Global Initiative for Asthma, 2020). AIT is an effective method for allergic diseases, with a history of 110 years since the first use in 1911. AIT can alleviate symptoms, change the allergic march, and still has a long-term effect when the treatment finished (Roberts et al., 2018). The emergence of biologics has provided a promising targeted therapy for asthma patients. These therapies have been shown to reduce asthma exacerbations and improve quality of life in appropriate patients (McGregor et al., 2019). With the approval of biologics such as omalizumab, dupilumab, benralizumab, mepolizumab, and reslizumab, they are more and more commonly used in patients with asthma or other allergic diseases. And the combination of biologics and AIT has been already explored in clinical practice.

However, although very rare, adverse effects especially anaphylaxis of AIT is still the problem that we face (Ryan et al., 2018). Bernstein et al. showed that the estimated frequency of very severe allergic reactions of SCIT was 1 in 2.5 million injection visits (Bernstein et al., 2004). Anaphylaxis in patients receiving omalizumab and reslizumab is also reported by post‐marketing surveillance, which ranges from 0.1 to 0.3% (Harrison et al., 2015; Cazzola et al., 2018).

Studies about anaphylaxis related to AIT and biologics in the real world are insufficient. As only omalizumab and dupilumab are available in China, we aimed in this study to analyze the anaphylactic reactions related to AIT, omalizumab, and dupilumab based on the US Food and Drug Administration Adverse Event Reporting System (FAERS).

## Materials and Methods

### Data Source

Using the FAERS database, a retrospective pharmacovigilance study was conducted from January 2004 to March 2021. The FAERS database is a public, voluntary, spontaneous reporting system (SRS) which contains adverse drug events and medication error reports submitted by health professionals, patients, and manufacturers from the United States and other countries. Seven types of datasets are included in the FAERS data files. It comprises patient demographic and administrative information (DEMO), drug information (DRUG), adverse events (REAC), patient outcomes (OUTC), report sources (RPSR), therapy start dates and end dates for reported drugs (THER), and indications for drug administration (INDI).

**FIGURE 1:**
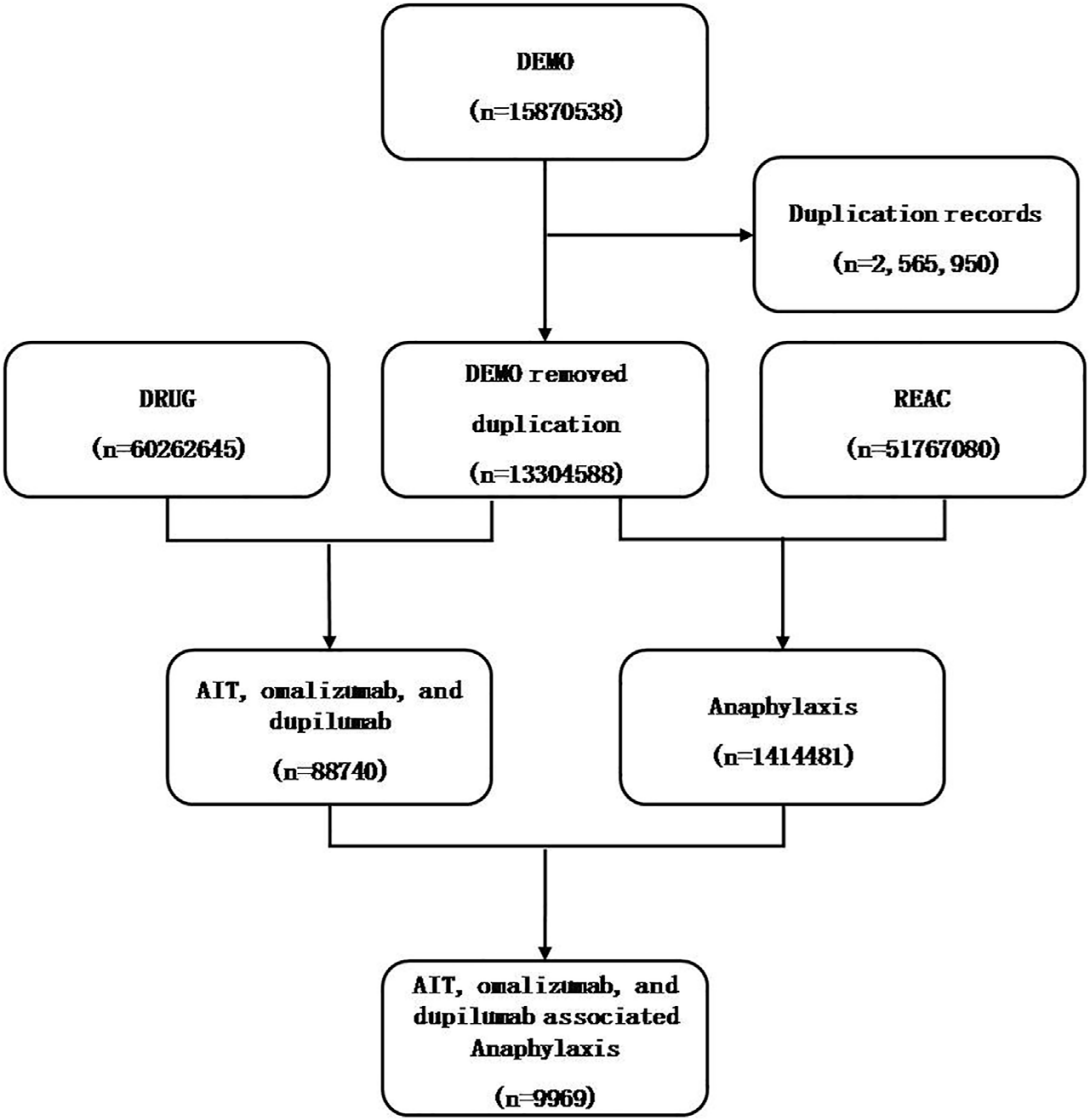
Flowchart of data mining process of anaphylaxis related to AIT, omalizumab, and dupilumab.

A total of 15,870,538 reports were got from the FAERS database. Then duplicated records were excluded according to the FDA recommendations. If the CASEIDs (number for identifying a FAERS case) were the same, the latest FDA_DT (date FDA received case) was selected. If the CASEID and FDA_DT were the same, the higher PRIMARYID (unique number for identifying a FAERS report) was selected. The final number was 9,969 (Figure 1). This study was approved by the institutional review board (IRB) of our hospital.

### Adverse Event and Drug Identification

According to Medical Dictionary for Regulatory Activities (MedDRA, version 22.1) at the Preferred Term level, anaphylactic symptoms were chosen from the REAC files. We considered the following preferred terms as related to anaphylactic symptom, especially in the scenario when AIT, omalizumab, and dupilumab were administered: “anaphylactic reaction (10002198)”, “anaphylaxis (10002218)”, “wheezing (10047924)”, “dyspnea (10013963)”, “cough (10011224)”, “respiratory distress (10038687)”, “hypoxemia (10021142)”, “stridor (10042241)”, “dysphonia (10013952)”, “throat tightness (10043528)”, “pharyngeal swelling (10082270)”, “abdominal pain (10000081)”, “vomiting (10047700)”, “diarrhea (10012727)”, “hypotension (10021097)”, “syncope (10042772)”, “loss of consciousness (10024855)”, “incontinence (10021639)”, “blood pressure decreased (10005734)”, with/without urticaria.

The AIT (including both subcutaneous immunotherapy and sublingual immunotherapy), omalizumab, and dupilumab’s generic and brand names were selected using IBM Micromedex as the dictionary during the data mining process.

### Data Mining

Depended on the primary principles of the Bayesian analysis and non-proportional analysis, the reporting odds ratio (ROR), proportional reporting ratio (PRR), Bayesian confidence propagation neural network, and multi-item gamma Poisson shrinker algorithms was adopted to identify the relation between the drug and the selected adverse events. The equations and criteria for each of the four algorithms are shown in Table 1(Evans et al., 2001; Szarfman et al., 2002; van Puijenbroek et al., 2002; Hauben, 2003; Hauben et al., 2005; Norén et al., 2006; Ooba and Kubota, 2010; Szumilas, 2010). We compared the association between anaphylactic reactions and different drugs. The given drug was considered as “primary suspect” in the ROLE_COD (code for the drug’s reported role in event) field of the DRUG files.

**TABLE 1:** Summary of major algorithms used for signal detection.

Algorithms	Equation^a^	Criteria
ROR	ROR = (a/b)/(c/d)	95% CI > 1, N ≥ 2
	95% CI = e^ln(ROR)±1.96(1/a+1/b+1/c+1/d)^0.5^	
PRR	PRR = [a/(a + c)]/[b/(b + d)]	PRR ≥ 2, χ^2^ ≥ 4, N ≥ 3
	χ^2^ = Σ [(O-E)2/E]; [O = a, E = (a + b) (a + c)/(a + b + c + d)]	
BCPNN	IC = log_2_a (a + b + c + d)/[(a + c) (a + b)]	IC025 > 0
	IC025 = e^ln(IC)−1.96(1/a + 1/b + 1/c + 1/d)^0.5^	
MGPS	EBGM = a (a + b + c + d)/[(a + c) (a + b)]	EBGM05 > 2, N > 0
	EBGM05 = e^ln(EBGM)−1.64 (1/a+1/b+1/c+1/d)^0.5^	

a a: number of reports containing both the suspect drug and the suspect adverse drug reaction. b: number of reports containing the suspect adverse drug reaction with other medications (except the drug of interest). c: number of reports containing the suspect drug with other adverse drug reactions (except the event of interest). d: number of reports containing other medications and other adverse drug reactions. Abbreviations: ROR, reporting odds ratio; CI, confidence interval; N, the number of co-occurrences; PRR, proportional reporting ratio; χ^2^, chi-squared; BCPNN, Bayesian confidence propagation neural network; IC, information component; IC025, the lower limit of the 95% two-sided CI of the IC; MGPS, multi-item gamma Poisson shrinker; EBGM, empirical Bayesian geometric mean; EBGM05, the lower 90% one-sided CI of EBGM.

The time to onset of the anaphylactic reaction for the different kinds of drugs was also estimated. It was defined as the interval between the EVENT_DT (adverse event onset date) and the START_DT (start date of the drugs administration). Records with wrong entry or incorrect inputs (EVETN_DT earlier than START_DT) were removed.

Furthermore, reports with fatal events due to anaphylactic drug reactions and the mortality rate were analyzed.

### Statistical Analysis

A descriptive analysis was used to describe the clinical characteristics of the cases with anaphylactic events due to AIT, omalizumab, and dupilumab from the FAERS database. The onset times of drug-associated anaphylactic symptoms among different drugs were compared using non-parametric tests (the Mann-Whitney U-test for dichotomous variables and the Kruskal-Wallis test when there were more than two subgroups of respondents). A pearson’s chi-squared test or Fisher’s exact test was used to compare the outcomes among different kinds of drugs. The statistical significance was set at *p* < 0.05 with 95% confidence intervals. All data mining and statistical analyses were conducted using SPSS, version 16.0 (SPSS Inc., Chicago, IL, United States).

## Results

### General Characteristics of all Reported Cases With Anaphylaxis Related to AIT, Omalizumab, and Dupilumab

In total, 9,969 reports of anaphylaxis related to AIT, omalizumab, and dupilumab were identified in the FAERS database from January 2004 to March 2021 (Flowchart is shown in Figure 1). The number of anaphylactic events related to AIT, omalizumab, and dupilumab were 64, 7,784, and 2,121, respectively. Omalizumab accounted for most of the reports (78%). Omalizumab associated anaphylaxis were more commonly reported in the recent 5 years (66%) than in the previous years. Anaphylactic events were more common in female (71 vs. 29%, *p* < 0.01), and children accounted for 7% of all reports. The demographic characteristics are shown in Table 2.

**TABLE 2:** Demographic characteristics of cases with AIT, omalizumab, and dupilumab associated anaphylaxis.

Characteristics	Reports (n)			
	AIT	Omalizumab	Dupilumab	Total
Age (years)				
<18	15	507	134	656
≥18	49	7,273	1,987	9,309
Unknown	0	4	0	4
Gender				
Female	34	5,338	881	6,253
Male	26	2,005	474	2,505
Unknown	4	441	766	1,211
Reporter				
Consumer	14	2,959	1,328	4,301
Lawyer	0	3	0	3
Other health-professional	19	1,036	111	1,166
Pharmacist	2	147	33	182
Physician	22	3,326	488	3,836
Unknown	7	313	161	481
Report year				
2004–2009	7	655	0	662
2010–2015	42	2,003	0	2,045
2016–2021	13	5,123	2,121	7,257
Unknown	2	3	0	5
Total	64	7,784	2,121	9,969

AIT: Allergen immunotherapy.

### Anaphylactic Events Associated With Different Drugs

Anaphylactic events were screened for AIT, omalizumab, and dupilumab depended on the criteria for the four algorithms (Table 3). Among these drugs, AIT had the highest ROR, PRR, information component (IC), and empirical Bayes geometric mean (EBGM), which was considered to be more related to anaphylaxis. Omalizumab showed a relatively lower ROR, while dupilumab had a negative signal for anaphylactic reaction.

**TABLE 3:** Comparison of anaphylaxis signals associated with AIT, omalizumab, and dupilumab.

	N	ROR (95%CI)	PRR (χ^2^)	IC (IC025)	EBGM (EBGM05)
AIT	64	5.03 (3.69, 6.85)	3.52 (129.22)	1.82 (1.33)	3.52 (2.72)
Omalizumab	7,784	2.24 (2.18, 2.29)	1.98 (4,179.78)	0.98 (0.95)	1.97 (1.93)
Dupilumab	2,121	0.36 (0.35, 0.38)	0.38 (2,300.84)	−1.37 (-)	0.39 (0.37)

ROR: reporting odds ratio; CI: confidence interval; PRR: proportional reporting ratio; χ2: chi-squared; IC: information component; IC025: the lower limit of the 95% two-sided CI of the IC; EBGM: empirical Bayesian geometric mean; EBGM05: the lower 90% one-sided CI of EBGM; AIT: allergen immunotherapy.

### General Characteristics of Cases Reporting Anaphylaxis Related to AIT, Omalizumab, and Dupilumab in Children

Among the 9,969 reports of anaphylaxis related to AIT, omalizumab, and dupilumab, 656 (7%) were children (under 18 years old). They were further divided into three age groups as 0–5, 6–11, and 12–17 years. Most anaphylactic reactions were reported in the 12– 17 year-old group (*n* = 446, 68%). More reports of anaphylaxis related to AIT were in boys (73%), while more reports of anaphylaxis related to omalizumab (63%) and dupilumab (58%) were in girls (Table 4). Asthma was the most common indication for use of omalizumab (69%), followed by chronic spontaneous urticaria (16%). Atopic dermatitis was the most common indication for use of dupilumab (57%), followed by asthma (30%) (Table 5).

**TABLE 4:** Demographic characteristics of cases with AIT, omalizumab, and dupilumab associated anaphylaxis in children.

Characteristics	Reports (n)			
	AIT	Omalizumab	Dupilumab	Total
Age (years)				
0–5 y	0	22	4	26
6–11 y	6	145	33	184
12–17 y	9	340	97	446
Total	15	507	134	656
Gender				
Girl	4	310	57	371
Boy	11	184	42	237
Unknown	0	13	35	48
Total	15	507	134	656

AIT: Allergen immunotherapy.

**TABLE 5:** Indications for the application of AIT, omalizumab, and dupilumab in children.

Indications	Reports (n)			
	AIT	Omalizumab	Dupilumab	Total
Anaphylactic reaction	0	2	0	2
Anti-allergic therapy	1	0	0	1
Asthma	0	347	35	382
Bronchospasm	0	1	0	1
Chronic spontaneous urticaria	0	79	0	79
Atopic Dermatitis	0	3	66	69
Food allergy	0	3	0	3
Hypersensitivity	3	1	0	4
Immune system disorder	1	1	0	2
Immune tolerance induction	3	0	0	3
Mast cell activation syndrome	0	1	0	1
Nasal polyps	0	0	11	11
Obstructive airways disorder	0	1	0	1
Allergic rhinitis	1	1	0	2
Seasonal allergy	2	0	0	2
Sinusitis	0	1	0	1
Skin test	0	1	0	1
Unknown	0	62	4	66
Total	11	504	116	631

AIT: Allergen immunotherapy.

### Time Interval Between Drug Initiation and Anaphylactic Symptoms in Children

**FIGURE 2:**
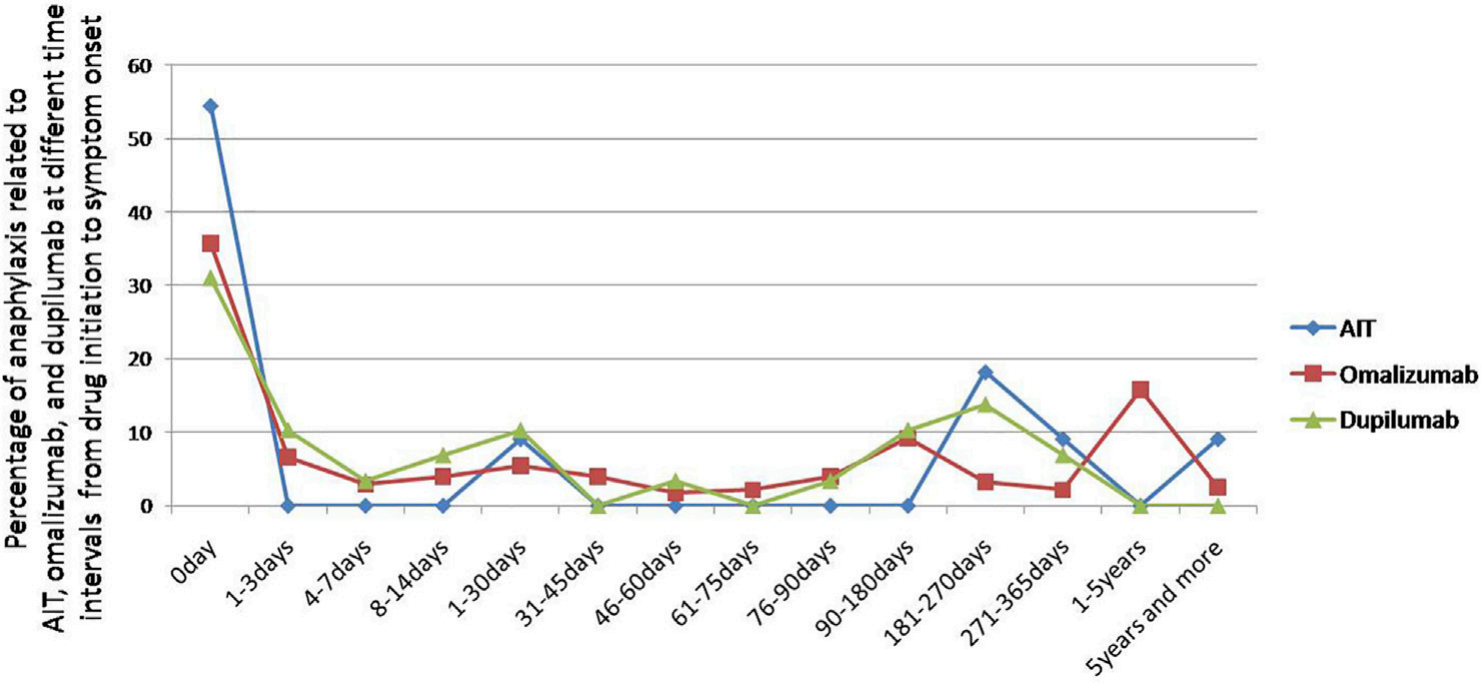
Percentage of anaphylaxis related to AIT, omalizumab, and dupilumab at different time intervals from drug initiation to symptom onset.

**FIGURE 3:**
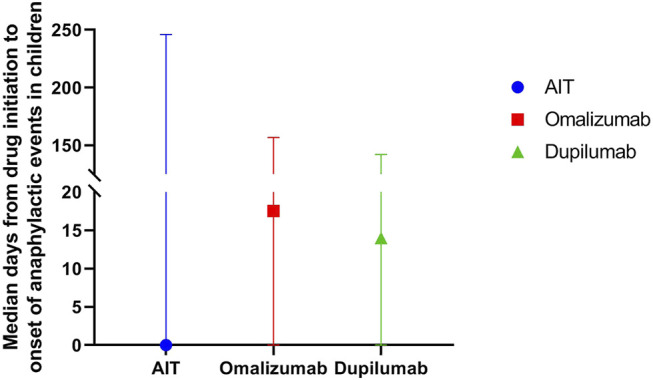
Median days (IQR) from drug initiation to onset of anaphylactic symptoms of AIT, omalizumab, and dupilumab in children.

Most symptoms occurred on the day of drug initiation, the percentage of anaphylaxis was small at seven and more days after drug initiation (Figure 2). The median day from drug initiation to onset of symptoms was 0 [interquartile range (IQR) 0–246] day, 17.5 (IQR 0–156.8) days, and 14 (IQR 0–142) days for AIT, omalizumab, and dupilumab, respectively (Figure 3). There was no significant difference among the three drugs (*p* > 0.05).

### Prognosis of Cases With AIT, Omalizumab, and Dupilumab Related Anaphylaxis in Children

We further analyzed the prognosis of cases with AIT, omalizumab, and dupilumab related anaphylaxis in children (Table 6). Death rate of AIT was the lowest (0), while death rate of dupilumab was the highest (4.76%). However, no differences were observed among the three drugs (*p* > 0.05). Hospitalization (initial or prolonged) rate and life-threatening rate of AIT were higer than omalizumab and dupilumab, differences among the three drugs was not significant (*p* > 0.05).

**TABLE 6:** Clinical outcome of cases with AIT, omalizumab, and dupilumab related anaphylaxis in children.

Outcome	Reports (n, %)		
	AIT	Omalizumab	Dupilumab
Death	0 (0.00)	4 (0.87)	2 (4.76)
Disability	0 (0.00)	4 (0.87)	1 (2.38)
Hospitalization-initial or prolonged	4 (33.3)	114 (24.68)	7 (16.67)
Life-threatening	1 (8.33)	22 (4.76)	0 (0.00)
Other serious (important medical event)	6 (50.00)	316 (68.40)	32 (76.19)
Required intervention to prevent permanent impairment/damage	1 (8.33)	2 (0.43)	0 (0.00)

AIT: Allergen immunotherapy.

## Discussion

In this study, we conducted a real-world study of anaphylactic events associated with AIT, omalizumab, and dupilumab based on FAERS. The clinical characteristics and outcome of reported cases particularly in children with anaphylaxis were described and ROR of anaphylactic reaction was analyzed. It may reflect the real-world condition in clinical practice. This study will give us more experience for application of these drugs which are usually prescribed in allergic patients.

Among all the reports of anaphylaxis related to AIT, omalizumab, and dupilumab, omalizumab associated anaphylaxis accounted for the most. Omalizumab was approved by FDA in 2003 and was widely used for nearly 20 years. While dupilumab was approved by FDA in March 2017, the using time was shorter than omalizumab. This might be one of the reasons why anaphylactic reports of omalizumab were more than dupilumab.

The anaphylactic symptoms of omalizumab and dupilumab were more common in female than in male. In another study of biologics related anaphylaxis based on FAERS, females made up a large part of reported cases (Li et al., 2021). A report of anaphylaxis associated with omalizumab also revealed a preponderance of female subjects (84%) (Lieberman et al., 2017). This reminded us that female might be a potential risk factor of biologics associated anaphylactic reaction. In children, AIT related anaphylaxis was more common in boys, omalizumab and dupilumab related anaphylaxis was more common in girls. We should be more cautious when boys were prescribed of AIT, and when girls were prescribed of omalizumab and dupilumab. However, as the number of anaphylactic reactions of AIT was small, this will be further analyzed in future studies.

The Bayesian analysis and non‐proportional analysis showed that AIT had the highest ROR, which was considered to be highly related to anaphylaxis. In a survey of near-fatal immunotherapy reactions from 1990 to 2001 conducted among member practices of the American Academy of Allergy, Asthma and Immunology, fatal reactions was estimated to occur every 1 per 2.5 million injections, with an average of 3.4 deaths per year. Among the fatal deaths, most were asthmatic patients who were not optimally controlled (Bernstein et al., 2004). Then from 2008 to 2012, among 23.3 million injection visits, subcutaneous allergen immunotherapy (SCIT)-related systemic allergic reactions (SRs) remained stable at 0.1% (Epstein et al., 2014). Later, data of 2013 was added and totally 28.9 million injection visits were gathered from 2008 to 2013. The rate of SRs from SCIT was 1.9%, with 0.08 and 0.02% of grade 3 and grade 4 SRs, respectively (Epstein et al., 2016). The newly updated data was from 2008 to 2018, with 64.5 million injection visits gathered. One fatal reaction occurred per 7.2 million injection visits. Ten confirmed fatalities occurred since 2008, including three new fatalities since 2017. SCIT-related fatalities have declined since 2008, with a slight increase in recent years (Epstein et al., 2021). Risk management should focus mainly on patients with uncontrolled asthma, with recent worsening in asthma symptoms and peak expiratory flow rate (Bernstein and Epstein, 2020). According to the above studies, the number of anaphylaxis was more than the number of this study, with the reason that some events might not be reported by the FAERS database. And this was also a limitation of the database.

Omalizumab had a positive signal for anaphylactic reaction and dupilumab had a negative signal for anaphylactic reaction. Omalizumab was reported of an anaphylactic rate of 0.1–0.2% (Cox et al., 2011), and FDA issued a black boxing warning for this drug. In the clinical trial of dupilumab in moderate-to-severe uncontrolled asthma, the most frequent adverse events were injection-site reaction and eosinophilia, and no anaphylaxis was reported (Castro et al., 2018). Reactions are less common with fully human biologics due to their lack of mouse antibody parts. However, immunogencity persists likely due to the use of transgenic mouse cell lines. Humanized biologics has 90% of human component with the generic suffix as “-zumab”, and fully human biologics has 99% of human component with the generic suffix as “-umab”. The more composition of human component, the lower potential of immunogenicity (Isabwe et al., 2018). Therefore, we should also be careful for these biologics. This study suggested the risk of anaphylaxis might be lower when AIT was combined with dupilumab than with omalizumab. Although biologics-AIT combination therapy is a valuable option treatment to improve both AIT safety and efficacy in widely variable scenarios of clinical risk, it seems that the use of biologics as add-on should be limited to those patients in whom a build-up escalation or maintenance dose can’t be tolerated, and where the use of AIT remains mandatory. Clinical trials are also needed to identify target patients, as well as optimal dosing schedules and duration of treatment, and better define cost-effectiveness (Malipiero et al., 2021).

In this study, most anaphylactic symptoms occurred on the day of drug initiation. In another national surveillance study of adverse reactions to allergen immunotherapy, nearly all fatal anaphylactic reactions and SRs occur within 30 min of injections. Delayed-onset SRs beginning over 30 min after injections accounted for 15% of all SRs. Therefore, a 30-min observation period is recommended (Bernstein and Epstein, 2020).

Death rate of AIT, omalizumab, and dupilumab associated anaphylaxis in children was low. Other studies also showed that the fatal reactions to AIT were low (Epstein et al., 2021). Death rate of omalizumab associated anaphylaxis was a little lower than that of dupilumab associated anaphylaxis, which was consistent with another real-world study about anaphylaxis related to biologis (Li et al., 2021). In a systematic review for the EAACI guidelines of recommendations on the use of biologics in severe asthma, omalizumab might increase serious adverse events in adolescent/adults (Risk ratio 1.62, 95%CI 0.76–3.45) with low certainty of evidence. No drug-related serious adverse events were reported for children of 6–11 years old (Agache et al., 2020).

This study also had some limitations. First, the total number of patients who received the treatments was unknown, therefore the rate of anaphylactic events for suspected drug couldn’t be analyzed. Second, the information of the cases reported was incomplete. Types of anaphylaxis couldn’t be analyzed. And the underlying diseases of the patients were unknown, which might impact on the outcome results. Third, this database was voluntarily reported by physicians, pharmacist, consumer, etc. Reporting behaviors might be influenced by recent publication of adverse events or FDA warning. These might lead to overestimate or underestimate of the results.

In conclusion, in this real-world study based on FAERS, AIT had the highest ROR for anaphylactic events, followed by omalizumab, and dupilumab had a negative signal for anaphylactic events. As well as AIT, patient should also be strictly monitored after administration of biologics. It also gives us a suggestion for choosing a combined biologics with AIT when the risk of anaphylaxis was considered.

## Data Availability

The original contributions presented in the study are included in the article/Supplementary Material, further inquiries can be directed to the corresponding authors.
